# A role for cyclooxygenase-1 in β-amyloid-induced neuroinflammation  

**DOI:** 10.18632/aging.100039

**Published:** 2009-04-13

**Authors:** Eduardo Candelario-Jalil

**Affiliations:** Department of Neurology, University of New Mexico, Health Sciences Center MSC10 5620, Albuquerque,; NM 87131-0001, USA

**Keywords:** β-amyloid, COX-1, microglia, neuroinflammation, Alzheimer's disease, oxidative stress, COX-2

Alzheimer's disease (AD) is a chronic neurodegenerative disorder characterized by progressive cognitive decline and memory loss. Accumulation of β-amyloid (Aβ) and tau protein are believed to be important pathological features of AD [1]. Results from a large number of studies suggest that neuroinflammation is a key contributor to neuronal loss in AD. Anti-inflammatory drugs, in particular non-steroidal anti-inflammatory drugs (NSAIDs), seem to be beneficial in terms of slowing the development of AD, as shown by several epidemiological studies [2-4]. Inhibition of cyclooxygenase (COX) activity is the main mechanism of action of NSAIDs. Two COX isoforms, COX-1 and COX-2, have been identified. Clinical studies evaluating the effects of NSAIDs or COX-2 selective inhibitors on AD have failed to show therapeutic efficacy. Some authors have suggested that the main reason for this is that COX-1 and COX-2 are involved in AD neuropathology in a preclinical stage of the disease. This explains the positive reports of the epidemiological studies and the negative findings in clinical trials with COX inhibitors [5,6]. Long-term use of NSAIDs might reduce the risk of AD, if the treatment starts before the onset of AD dementia [7].

There is considerable debate on the relative contribution of each COX isoform to AD  pathology.  In AD brains, neuronal COX-2 levels have been found to be either elevated in early stages [8-10] or decreased in end-stage [11]. It is interesting to note that an upregulation in early AD and reduction of COX-2 in advanced AD correlates very nicely with the levels of prostaglandin E_2_ (PGE_2_) in the CSF, which are increased in subjects with mild memory impairment (probable AD diagnosis) and decreased with increasing severity of AD dementia [12,13]. COX-2 is expressed in neurons, but not in astrocytes or microglia in AD brains [5]. Transgenic mice in which human COX-2 is overexpressed constitutively in neurons develop age-dependent cognitive deficits that are associated with a parallel age-dependent increase in neuronal apoptosis and astrocytic activation [14]. Overexpression of COX-2 in APPswe-PS1dE9 mice leads to age-dependent cognitive deficits in females but not male mice, without significantly affecting Aβ accumulation. The cognitive deficits in female COX-2/APPswe-PS1dE9 mice are reversed with administration of the COX-2 selective inhibitor celecoxib [15]. This suggests a sex-dimorphic involvement of COX-2 in AD neuropathology. Selective inhibition of COX-2, but not COX-1, prevented the suppression of hippocampal long-term potentiation (LTP) induced by Aβ_1-42_. The NSAIDs, ibuprofen and naproxen, and a selective COX-2 inhibitor restored memory function in Tg2576 mice overexpressing APP [16]. Interestingly, COX-1-expressing microglia surrounds amyloid plaques [17]. There is no evidence that COX-1 expression in microglia is changed in AD brain [5]. However, accumulation of COX-1-expressing microglia in AD could result in local increase in prostaglandin synthesis and oxidative stress.

In a very recent article by Choi and Bosetti, published in the February issue of *Aging*, they report for the first time the effect of COX-1 gene deletion on the neurotoxicity associated with Aβ[18]. These data provide strong experimental evidence linking COX-1 activity to neuronal loss following intracerebroventricular administration of Aβ. Authors found a dramatic inflammatory response within the CA1 and CA3 areas of the hippocampus in 3-month-old wild-type mice seven days after Aβ_1-42 _peptide injection. This neuroinflammatory response was characterized by the presence of Iba-1-positive activated microglia, increased GFAP-immunoreactive astrocytes, and elevated oxidative stress markers. Interestingly, COX-1 deficient mice displayed a significant reduction in the number of activated microglia in the CA3 region of the hippocampus as well as in the number of GFAP-positive reactive astrocytes, indicating that Aβ_1-42_ injection induced less severe glial activation in COX-1 knockout animals compared to wild-type control mice. In addition, COX-1 deficiency was associated with decreased oxidative damage, suggesting that enhanced COX-1 activity is a significant source of oxidative stress in Aβ-mediated neurotoxicity. Levels of PGE_2_, PGF_2__α_and thromboxane B_2_ (TXB_2_) were significantly reduced in COX-1 null mice compared with wild-type controls. More importantly, COX-1 deletion resulted in reduced neuronal damage following Aβ_1-42 _admi-nistration, as shown by a reduced number of Fluoro-Jade B (FJB)-positive cells in the hippocampus.

The classical view that COX-2 is more important than COX-1 in neuroinflammatory processes should be revisited. The data of Choi and Bosetti [18], together with findings from other studies [19-21] indicate that COX-1 is actively involved in brain injury induced by pro-inflammatory stimuli including Aβ, lipopolysaccharide (LPS) and TNF-α. In some models of neuroinflammation, COX-2 deletion or pharmacological inhibition with selective agents exacerbate rather than reduce inflammation-related brain damage [22]. COX-1 is prominently expressed by microglia [8,17]. Due to the key role of microglia in neuroinflammation, it has been suggested that selective inhibition of COX-1, rather than COX-2, will be more effective in treating neuroinflammation and neurodegeneration [23].

Reduction in cognitive decline in AD patients was observed in a 6-month, double-blinded, placebocontrolled study with indomethacin, a non-selective, but a potent COX-1 inhibitor [24]. No beneficial effects were observed with the COX-2 selective inhibitors celecoxib and rofecoxib [25-29]. Based on these previous studies and their own data [18], Choi and Bosetti propose the intriguing hypothesis that the potential protective effects of NSAIDs in AD may be related to COX-1, but not COX-2 inhibition. In support of this notion, a previous study showed that neurons treated with COX-1 selective inhibitors are resistant to Aβ_1-42 _[30]. Moreover, COX-1 inhibition produced a profound inhibition of either LPS- or arachidonic acid-induced PGE_2_ synthesis in human microglia [31].  However, with the exception of one small pilot study [24], no therapeutic efficacy in AD clinical trials have been found with NSAIDs, including nonselective inhibitors such as naproxen and diclofenac, which inhibit both COX-1 and COX-2 [5,26].

A major limitation of chronic COX-1 inhibitor treatment of AD patients is the gastrointestinal toxicity, due to the suppression of COX-1-mediated production of protective prostaglandins. In the pilot clinical study showing beneficial effects of indomethacin in AD patients, the dropout rate in the indomethacin group was approximately 40%, mostly due to drug-related gastrointestinal adverse events  [24]. There is obviously a risk attached to taking any drug. However, clinical demonstration of a positive benefit/risk ratio of NSAIDs in AD patients is missing. It has been questioned whether anti-inflammatory interventions are really a viable or even preventive option for AD [6].

It is still debatable whether targeting neuroinflammatory events in AD, primarily microglial activation, is a promising therapeutic option. In AD, microglia accumulate in senile plaques and may have a dual role, either digesting or contributing to the formation of Aβ plaques [1]. The idea of removal of senile plaque constituents by microglia was first proposed by Timmer in 1925, who suggested that these cells were mobilized to phagocytose toxic products and formed the core of senile plaques [32]. Clusters of microglial cells with rounded and phagocytic phenotypes are found in fibrillar Aβ deposits in the neocortex of AD brain [33]. In an elegant study published in *Neuron*, Simard and colleagues showed that it is the bone marrow-derived microglia, and not their counterparts resident in the brain, that have the ability to promote the clearance and phagocytosis of Aβ[34]. On the other hand, the production of inflammatory mediators by microglia might contribute to the formation of Aβ plaques [35]. Therefore, the specific role of these cells in the evolution of the senile plaques in AD is still under debate.

Early accumulation of microglial cells in AD delays disease progression by facilitating clearance of Aβ before formation of senile plaques. However, persistent Aβ accumulation despite increasing microglial numbers indicate that the capacity of microglia to phagocytose Aβ may be impaired with age. A recent study indicates that the progression of Aβ plaque formation is associated with an aging-dependent dysfunction of microglia in the PS1-APP transgenic mice, an AD mouse model [36]. The number of senile-like plaques and microglia associated with these plaques are increased as the mice age. Microglia from old PS1-APP mice, but not from younger mice, have a decrease capacity to clear Aβ. This may be related to the increased production of proinflammatory mediators and to a downregulation of genes involved in Aβ removal [36]. These results illustrate the dichotomous role of microglia in AD pathology.

Data from the report by Choi and Bosetti [18] are exciting and have potential clinical implications. This study will fuel future experimentation to elucidate the specific role of each COX isoform in neuroinflammation and neurodegenerative processes. It remains to be determined how COX-1 inhibition modifies the early beneficial function of activated microglia in Aβ clearance and whether COX-1 inhibition is protective against neuronal loss in other models of AD such as the PS1-APP transgenic mice. Is COX-1 inhibition also protective in older animals and females? Future research will definitely provide answers to these questions. Elucidating the role of neuroinflammatory events in AD may provide oppor-tunities toward the development of new therapeutic strategies to tackle neurodegeneration associated with AD and possibly other neurodegenerative disorders.
